# HLA Genotypes and Type 1 Diabetes and Its Relationship to Reported Race/Skin Color in Their Relatives: A Brazilian Multicenter Study

**DOI:** 10.3390/genes13060972

**Published:** 2022-05-29

**Authors:** Marília B. Gomes, Luís C. Porto, Dayse A. Silva, Carlos A. Negrato, Elizabeth João Pavin, Renan Montenegro Junior, Sergio A. Dib, João S. Felício, Deborah C. Santos, Luiza H. Muniz, Rosângela Réa, Rossana Sousa Azulay, Vandilson Rodrigues

**Affiliations:** 1 Diabetes Unit, Rio de Janeiro State University, Rio de Janeiro 20551-30, Brazil; deborahconte@hotmail.com (D.C.S.); luizaharcarmuniz@gmail.com (L.H.M.); 2 Histocompatibility and Cryopreservation Laboratory, Rio de Janeiro State University, Rio de Janeiro 20950-003, Brazil; lcporto@uerj.br; 3 DNA Diagnostic Laboratory, Rio de Janeiro State University, Rio de Janeiro 20550-900, Brazil; dayse.a.silva@gmail.com; 4 Medical Doctor Program, University of São Paulo, Bauru 17012-230, Brazil; carlosnegrato@uol.com.br; 5 Endocrinology Division, Internal Medicine Department, School of Medical Sciences, State University of Campinas, Campinas 13083-887, Brazil; ejpavin@gmail.com; 6 Department of Community Health, Federal University of Ceará, Fortaleza 60430-235, Brazil; renanmmjr@gmail.com; 7 Department of Medicine, Federal University of São Paulo, São Paulo 04023-062, Brazil; sergio.dib@unifesp.br; 8 Endocrinology Division, Federal University of Pará, Belém 66050-060, Brazil; felicio.bel@terra.com.br; 9 Department of Internal Medicine, Federal University of Paraná, Curitiba 80060-900, Brazil; rosangelarea@uol.com.br; 10 Service of Endocrinology, University Hospital of the Federal University of Maranhão, São Luís 65020-070, Brazil; rossanaendocrino@gmail.com; 11 Research Group in Clinical and Molecular Endocrinology and Metabology, Federal University of Maranhão, São Luís 65080-805, Brazil; vandilson.rodrigues@ufma.br

**Keywords:** HLA, relatives, reported race/skin color, type 1 diabetes, ethnicity

## Abstract

We aimed to investigate the relationship between HLA alleles in patients with type 1 diabetes from an admixed population and the reported race/skin color of their relatives. This cross-sectional, multicenter study was conducted in public clinics in nine Brazilian cities and included 662 patients with type 1 diabetes and their relatives. Demographic data for patients and information on the race/skin color and birthplace of their relatives were obtained. Typing of the HLA-DRB1, -DQA1, and -DQB1 genes was performed. Most studied patients reported having a White relative (95.17%), and the most frequently observed allele among them was DRB1*03:01. Increased odds of presenting this allele were found only in those patients who reported having all White relatives. Considering that most of the patients reported having a White relative and that the most frequent observed allele was DRB1*03:01 (probably a European-derived allele), regardless of the race/skin color of their relatives, we conclude that the type 1 diabetes genotype comes probably from European, Caucasian ethnicity. However, future studies with other ancestry markers are needed to fill the knowledge gap regarding the genetic origin of the type 1 diabetes genotype in admixed populations such as the Brazilian.

## 1. Introduction

Type 1 diabetes (T1D) is a chronic autoimmune disease resulting from an interplay between genetic predisposition and multiple environmental factors [1]. A steep rise in the incidence of T1D has been observed worldwide, including in Brazil [2,3]. According to the International Diabetes Federation [2], the highest incidence is found in Caucasian populations (Finland and Sweden), although other ethnicities have also shown an increase in the number of individuals with T1D in their populations.

So far, the most important T1D genetic markers are the alleles of the histocompatibility leukocyte antigen system (HLA) region located on chromosome 6p21.3, which are involved in human immune response [1]. Although class I alleles and non-HLA alleles also contribute to T1D risk, class II (*HLA-DRB1*, *-DQA1* and *DQB1*) alleles are the most important and account for almost 50% of the genetic risk for this disease [4]. The haplotypes DRB1*04~DQA1*03:01~DQB1*03:02[DR4-DQ8] and DRB1*03:01~DQA1*05:01~DQB1*02:01[DR3-DQ2] [5] are associated with higher genetic risk for the disease. However, the frequency of these HLA haplotypes, and their effects on T1D risk or protection, vary among populations with different ethnicities [6]. For instance, the haplotype DRB1*07:01~DQA1*03:01~DQB1*02:02 appears to be associated with increased risk for African-Americans ^6^; in contrast, a change to the DQA1*02:01 allele in this haplotype is known to be protective for European populations ^1^. In addition, DRB1*07:01 has been found to have a protective effect in Brazilian individuals with T1D [1,7] and is also one of the most frequently observed alleles in the Brazilian population [8]. Based on these data, we hypothesize that the relationship between some HLA system alleles and ethnicity, as determined by self-reported race/skin color [7,9] or genomic ancestry [10,11], may contribute to T1D risk. Some methodologies allow the inference of genomic ancestry, like the autosomal ancestry-informative insertion deletion markers (AIM-INDELs), which provide more knowledge on genes’ structure; however, its high cost makes its use unfeasible in routine clinical practice [11].

Brazilian population has an important diversity and heterogeneous ancestry because it is primarily formed from three ancestral roots that are highly admixed: Native Americans, Europeans, and Sub-Saharan Africans [12]. Miscegenation among these 3 roots has occurred over centuries, resulting from asymmetric mating patterns, mainly between European men and Native American or African women [12]. Since 1991, self-reported race/skin color has been used for Brazilian population censuses and is divided into five groups: White (*branca*), Black (*preta*), Brown (*parda*), Yellow (*amarela*), and Indigenous (*indígena*) [13]. Considering the above-mentioned, the evaluation of reported race/skin color of patients’ relatives could increase the understanding of the genetic background of our population admixture and the genetic origin of T1D in admixed populations like ours.

The present study aimed to investigate the relationship between HLA genotypes and ethnicity in Brazilian patients with T1D, belonging to an admixed population and the reported race/skin color of their relatives. Our hypothesis is that there is a relationship between HLA haplotypes and alleles in patients with T1D with the reported race/skin-color and the birthplace of their relatives up to the third generation.

## 2. Methods

### 2.1. Study Design and Sample

This study included 662 patients with type 1 diabetes, who participated in a nationwide multicenter cross-sectional study. The study originally enrolled 1912 patients, and was conducted from August 2011 to August 2014 in nine public clinics, located in nine Brazilian cities from five geographical regions (North, Northeast, Midwest, Southeast, and South). The initial sample size was estimated based on the Brazilian Multicenter Diabetes Study described elsewhere [14], to maintain the representativeness of the distribution of T1D cases across each Brazilian geographic region according to the Brazilian census data [13]. Thus, the patients were initially screened by HLA data and by reporting relatives’ data up to the third generation. The overview of the study sample is presented in the Supplementary Figure S1. Briefly, all patients with T1D received health care through the Brazilian National Health Care System (BNHCS), known by the acronym of SUS (*Serviço Único de Saúde*), and had been diagnosed as having T1D based on the presence of typical clinical characteristics including variable degrees of hyperglycemia, weight loss, polyuria, polydipsia, polyphagia, and the need for continuous use of insulin since the diagnosis ^14^. Included patients had to be at least 13 years old and be followed at each respective diabetes center for a minimum of six months. Each respective diabetes center provided data from at least 50 outpatients with T1D who regularly attended the clinic.

This study was approved by the Ethic Committee of Pedro Ernesto University Hospital (Rio de Janeiro State University, number 1.440.365). Written informed consent was obtained from all patients or their parents where necessary.

### 2.2. Data Collection

Participants completed a standardized questionnaire during a clinical visit, which evaluated clinical and demographic data such as gender, current age, birthplace, self-reported race/skin color, age at diagnosis, and diabetes duration.

Information about the family history, including birthplace and relatives’ reported race/skin color, was obtained in detailed interviews with the participants or a family member, and data covered three generations (1st degree relatives = parents, 2nd degree relatives = grandparents, 3rd degree = great-grandparents). Reported race/skin color was evaluated based on the IBGE (Brazilian Institute of Geography and Statistics) classifications: Black skin color = *preta,* White skin color = *branca*, Brown skin color = *parda*, Yellow (Asian origin) = *amarela*, and Indigenous (Amerindian origin) = *indígena* [13].

### 2.3. HLA Typing

DNA extraction was performed on a peripheral blood sample using the SP QIA Symphony commercial kit according to the manufacturer’s guidelines (Qiagen, Germantown, MD, USA). HLA-class II alleles (*DRB1*, *DQA1*, and *DQB1*) from 1544 individuals with T1D were genotyped. A total of 476 (30.8%) participants with T1D had their DNA samples analyzed by Next Generation Sequencing (NGS). Of these, 352 were amplified at loci *HLA- DRB1* and *-DQB1* by long-range PCR using primers from the NGSgo^®^ v2 (GenDx, Utrecht, The Netherlands) library preparation kit, and 124 with the Holotype HLA Assay (Omixon Inc., Budapest, Hungary) for *HLA-DRB1*, *-DQB1* and *-DQA1*, according to the manufacturer’s instructions. Typification of the *HLA-DRB1*, *-DQA1*, and *-DQB1* genes was performed with the PCR–RSSO (high-resolution LabTypt (One Lambda Inc., West Hills, MI, USA), combined with Luminex technology for the 1069 other samples; these primers cover exons 2, 3, and 4. A catalog of common, intermediate and well-documented (CIWD) was used to define alleles, and ambiguities were solved by the sequencing method [15]. Three-locus haplotype frequencies (*DRB1~DQA1~DQB1*) were estimated for each reported race/skin color and region, resolving phase and allelic ambiguity using the expectation-maximization (EM) algorithm. Deviations from Hardy-Weinberg equilibrium (HWE) were assessed at the allele-family level (first nomenclature field) using a modified version of the Guo and Thompson algorithm [16], as implemented in the software Arlequin v.3.5 [17]. Pairwise linkage disequilibrium was also determined using Arlequin 3.5 for all three *loci*. *HLA-DQA1* alleles were imputed in 31.5% of the samples from individuals with T1D (*n* = 321) using the linkage disequilibrium criteria, based on the results found by NGS. Ambiguous HLA class II alleles within P or G groups were designated by a lower case ‘g’ (DRB1*12:01g = *12:01/12:10*; DQA1*01:01g = *01:01/01:04/01:05*; DQA1*03:01g = 03:01/03:02/03:03; DQA1*05:01g = *05:05/05:09*; DQB1*03:01g = *03:01/03:09/03:19*). After validating the HLA dataset via an EM algorithm for resolving allelic ambiguities and determining both allele and extended haplotype frequencies despite some missing loci data, this imputation was manually performed according to the haplotype results from Arlequin output data, according to reported race/skin color and region.

Due to the large number of alleles found, the table containing these alleles shows those with the total number greater than 1%. Infrequently found alleles are presented as “other” (≤1%).

### 2.4. Statistical Analysis

Data analysis was performed using SPSS software version 27.0 (IBM, Chicago, IL, USA) and GraphPad Prism software version 9 (GraphPad Software, San Diego, CA, USA). Descriptive statistics was performed to summarize the collected data. Data were stratified by kinship degree (1st, 2nd, and 3rd degree relatives) and birthplace of parents, grandparents, and great-grandparents. Reported birthplaces were then classified as Iberian Peninsula, Italy, Caucasus region, Japan, Middle East, and Africa, given the genetic proximity in these population groups [18,19].

For the analysis of association with specific alleles, samples were classified according to the identification of genotypes carrying HLA-DRB1*03 or/and -DRB1*04 allelic group. A multiple logistic regression analysis was performed to investigate predictors of *HLA-DRB1* alleles (alleles with frequency higher than 2% in the sample), to estimate adjusted odds ratio (OR) and 95% confidence interval (95% CI). In addition, principal components analysis (PCA) was conducted to explore the interrelationships between *HLA-DRB1* alleles in genotype, relatives’ reported race/skin color (all White, all Black, any Indigenous, any Yellow), and relatives born in Europe. Results were presented in tables, biplots, and bar charts. The level of significance adopted for all analyses was 5%.

## 3. Results

### 3.1. Overview of Demographic Data and HLA Allele Frequencies of the Studied Population with Type 1 Diabetes

The study included 662 patients. Demographic data of the studied population are described in Table 1. Most patients self-reported as being White (53.63%) or Brown (38.52%).

Supplementary Table S1 presents the reported race/skin color for patients’ relatives. Data showed that the most frequently reported race/skin colors were White and Brown for both maternal and paternal relatives. Most patients reported having at least one White relative in any degree (95.17%). Only nine (4.83%) patients reported all degree relatives as being Black. The birthplace distribution data from 1st (parents), 2nd (grandparents) and 3rd (great-grandparents) degree relatives and male/female relatives are shown in Supplementary Table S2.

**Figure 1:**
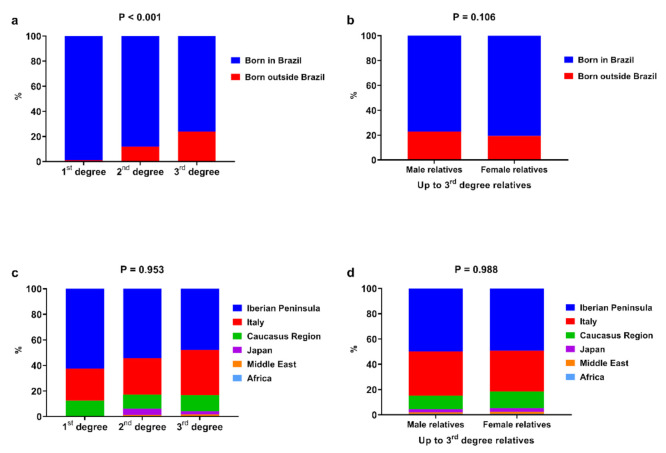
Comparative analysis of family birthplace according to the degree of kindship (**a**,**c**) and male/female relatives (**b**,**d**). Chi-square test was performed for statistical analysis.

Most of the relatives were born in Brazil. An increase in relatives born outside of Brazil was noted from the 1st to 3rd generation: 1.21%, 11.78%, and 24.02% for the 1st, 2nd, and 3rd generation, respectively (Figure 1a, *p* < 0.001). There was no difference between gender among the groups (Figure 1b, *p* = 0.106). The majority of the relatives born outside of Brazil were from the Iberian Peninsula and Italy (Figure 1c,d).

The HLA allele distribution is shown in Table 2. The most frequently observed *HLA-DRB1* allelic groups were -DRB1*03 (30.21%), -DRB1*04 (29.91%), -DRB1*01 (8.31%), and -DRB1*07 (8.23%). In addition, the most frequently observed *HLA-DQA1* allelic groups were -DQA1*05 (34.64%) and -DQA1*03 (34.19%), and for *HLA-DQB1* alleles were -DQB1*02 (39.50%) and -DQB1*03 (37.31%). Overall, the most frequently observed alleles were HLA-DRB1*03:01 (28.78%), HLA-DQA1*05:01 (34.27%) and HLA-DQB1*03:02 (29.15%). These data are described in Supplementary Tables S3–S5. A linkage disequilibrium was observed between *HLA-DRB1* and HLA-*DQA1*, *HLA-DRB1* and HLA-*DQB1*, and also *HLA-DQA1* and -*DQB1* (*p* < 0.001).

Supplementary Table S6 shows the frequencies of HLA-DRB1*/DRB1* genotype. Genotypes DRB1*03/DRB1*04 (23.56%), DRB1*03/DRB1*03 (8.76%), DRB1*04/DRB1*04 (6.65%), and DRB1*01/DRB1*04 (5.89%) presented the highest frequencies in the sample. 

### 3.2. Overview of the Association between HLA Alleles and Reported Race/Skin Color of the Relatives

**Figure 2:**
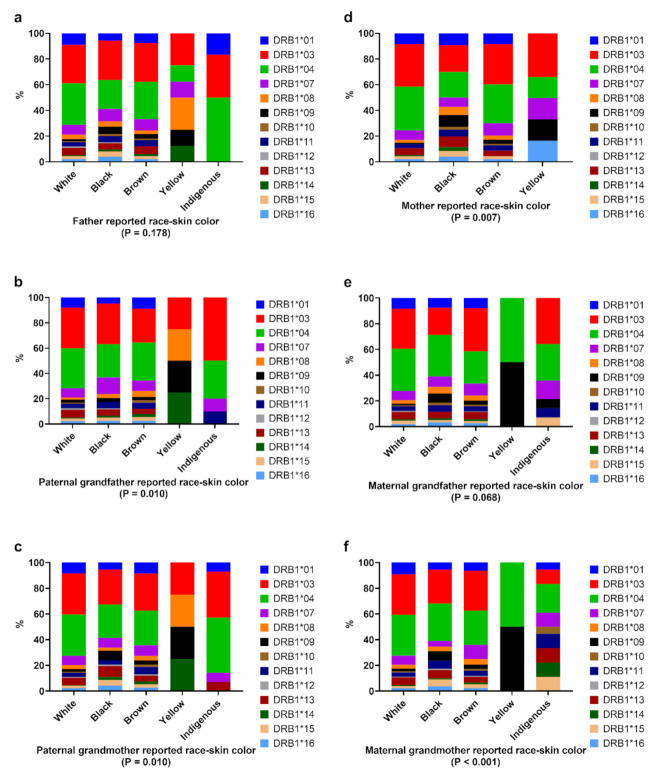
Distribution of HLA-DRB1 alleles according to reported race/skin color from paternal relatives (**a**–**c**) and maternal relatives (**d**–**f**). Chi-square test was performed for statistical analysis.

The association between *HLA-DRB1* alleles and relatives’ reported race/skin color is shown in Figure 2. There were significant differences in *HLA-DRB1* allele distribution according to the reported race/skin color of the mother (*p* = 0.007), paternal grandfather (*p* = 0.01), paternal grandmother (*p* = 0.01), and maternal grandmother (*p* < 0.001). The data highlight the higher frequency of HLA-DRB1*03 and -DRB1*04 in the White and Brown categories than in the Black category. There were no significant differences between reported race/skin color of the relatives and *HLA-DQA1* (Supplementary Figure S2) and *HLA-DQB1* (Supplementary Figure S3).

**Figure 3:**
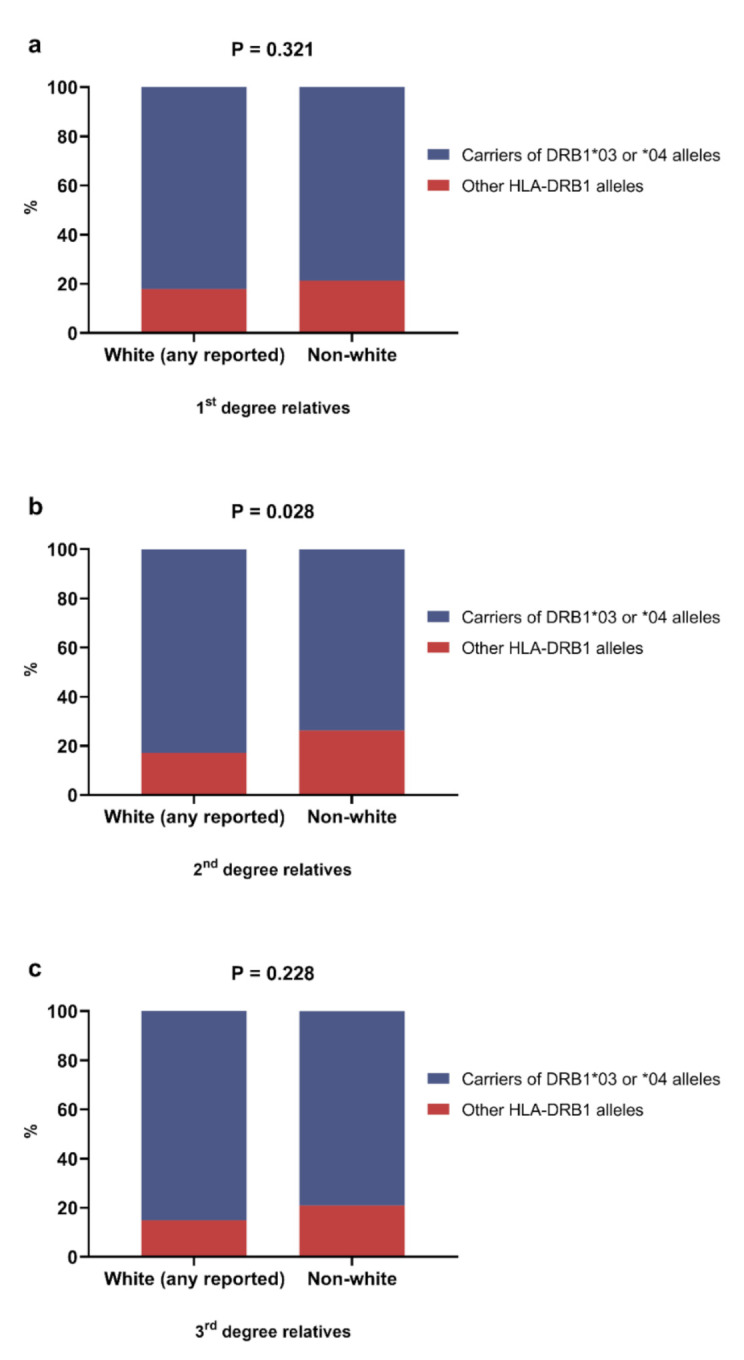
Distribution of carriers of HLA-DRB1*03 or/and 04 alleles according to reported race/skin color in 1st degree (**a**), 2nd degree (**b**) and 3rd degree (**c**) relatives. Chi-square test was performed for statistical analysis.

A higher frequency of patients carrying DRB1*03 and/or -DRB1*04 was observed in those who reported any White grandparent (*p* = 0.028) (Figure 3). There was no difference in the distribution of *HLA-DRB1* alleles (Supplementary Figure S4) or HLA-DRB1*03 or/and -DRB1*04 carriers according to the birthplace of the patients’ relatives (Supplementary Figure S5).

Multiple logistic regression analysis showed that patients who reported all parents, grandparents, and great-grandparents as being White had increased odds of being HLA-DRB1*03 carriers and decrease odds of being HLA-DRB1*07, -DRB1*09, and -DRB1*11 carriers. In addition, patients that reported any relatives as being Yellow had increased odds of presenting HLA-DRB1*09 (Table 3).

The principal component analysis on allele frequencies showed a clear cluster for all relatives reported as being White (Supplementary Figure S6a), and any relative reported as Yellow (Supplementary Figure S6d).

The most frequently observed *HLA-DRB1* genotypes in patients who reported all parents, grandparents, and great-grandparents as being White (*n* = 164) were -DRB1*03:01/03:01 (9.76%), -DRB1*03:01/04:02 (9.15%), and -DRB1*03:01/04:04 (7.93%). In this group, the -DRB1*04:01 allele in homozygous or heterozygous genotypes were present in 14.64% of the patients. The -DRB1*08, *09 and *10 alleles were only identified in patients carrying DRB1*01, *03 and *04 allelic groups (Table 4).

A total of 186 patients (28.09%) reported any degree relative as being Black (Supplementary Table S7), and 73.65% reported at least one relative as being White. In this group, a higher frequency of patients carrying the -DRB1*03:01 allele in homozygous or heterozygous genotypes was observed (42.52%). The genotype combination -DRB1*03:01/03:01 followed by -DRB1*03:01/04:04 was the most frequently found. Patients who reported only Black relatives up to the 3rd generation (*n* = 9, 4.83%) had no predominance of any DRB1 genotype (Supplementary Table S7).

Supplementary Table S8 shows the distribution of DRB1 genotypes in patients who reported any relative as being Indigenous (*n* = 38, 5.74%), or any relative as being Yellow (*n* = 8, 1.21%). There were no predominant DRB1 genotypes in patients who reported any relative as being Yellow, and all of them reported at least one White relative. In this latter group, three out of eight patients carried the allele -DRB1*09:01 haplotype.

The three most frequent HLA–DRB1*~DQA1*~DQB1* haplotypes observed in patients were 03:01~05:01g~02:01 (16.54%), 04:05~03:01g~03:02 (5.44%), and 03:01~03:01g~02:01 (5.36%) without statistical significance according to race/skin color of the relative’s (*p* < 0.1) (Supplementary Table S9).

## 4. Discussion

To the best of our knowledge, this is the first study relating HLA class II genotyping in individuals with T1D in a highly admixed population considering demographic data of relatives up to the 3rd generation. The present study showed that the most frequently observed alleles in patients with T1D were from locus HLA-DRB1, DRB1*01, DRB1*03, DRB1*04, DRB1* 09, and for the locus HLA-DQB1, -DQB1*02 and -DQB1*03. Alleles observed less frequently in patients with T1D were DRB1*07, DRB1*11 and DRB1*13.

All these alleles had also been previously described as risk alleles and/or protective for T1D in a multicenter study conducted in all geographic regions of Brazil [7], and also in some other countries [1,20]. It is important to emphasize that all these risk alleles had already been described in a meta-analysis conducted in Latin America with patients having T1D. However, in this meta-analysis, the allele DRB1*07 was not referred as protective, and this allele is one of the most frequently found in the Brazilian general population [8]. This fact highlights the genetic diversity found in Latin America.

The great majority of the relatives of both genders were reported as being White, followed by Brown. These findings showed that patients who reported all relatives up to the 3rd generation as being White had increased odds of being a HLA-DRB1*03 carrier and decreased odds of being a HLA-DRB1*07, -DRB1*09, and -DRB1*11 carrier. Although most relatives were born in Brazil, the number of relatives born outside of Brazil increased from the 1st to the 3rd generation, with relatives coming mainly from the Iberian Peninsula and Italy, reflecting the history of Brazilian colonization. However, no association between HLA system alleles and relatives’ birthplace was found.

We have found an association between the previously described patients’ HLA system risk alleles, DRB1*03, with the reported race/skin color of the relatives, mainly among those who reported all relatives as being White. It is important to note that only a small number of patients reported all relatives as being Black, and no one reported all relatives as being Indigenous or Yellow, reflecting the admixture observed in this population [12].

Brazil is the fifth largest country in the world, and has an estimated population of 210 million people, being 85.43% urban dwellers. The country was discovered by Portuguese navigators and was originally inhabited by Native Amerindians. Ever since, the Portuguese-Amerindian admixture started. In the 16th century, African slaves were brought to the country, and in the 17th and 18th centuries, Europeans came mostly as immigrants [21]. The Brazilian population has a high degree of genetic diversity as a result of five centuries of interethnic admixture among these three ethnic roots, and is considered one of the most admixed populations in the world [22,23]. Our data concerning relatives of patients with T1D align with the aforementioned data. Although most relatives were reported as being White, followed by Brown (probably resulting from an admixture between White and Black individuals), only 6.5% of patients self-reported as being Black, independently of their paternal or maternal lineage.

It is important to emphasize that our sample of relatives (three generations) covered only the previous 75 years, comprising only part of the period known as the “whitening of Brazil” (1872–1975), when approximately five million immigrants, mainly Italians and Iberians, came to live in this country [21,23]. Considering that each generation comprises a period of 25 years, we would have to study more ancestral generations to have a broader picture (representation) of the relationship between European immigration and the frequency of HLA system alleles found in patients with T1D in Brazil [12,21]. The present findings show that HLA-DRB1 allele frequencies were associated with the reported race-skin color of relatives; however, there was no particular trend from the 1st to the 3rd generation. The gene flow from Europeans could have occurred before the 3rd generation (great-grandparents) that was analyzed in the present study. This possibility is supported by the increase, from the 1st to the 3rd generation, in the number of relatives reporting as being born outside of Brazil, mainly in the Iberian Peninsula and Italy.

We found an association between T1D patients’ HLA risk alleles HLA-DRB1*03, previously described in a Brazilian Multicenter study [7], and the reported race/skin color of their matrilineal or patrilineal relatives, which was stronger for patients who reported all relatives as being White. Those patients who reported all relatives as being White presented a decrease in the odds ratio of being HLA-DRB1*07 or -DRB1*11 T1D, as previously described in this Brazilian multicenter study, as well as in other studies as protective alleles for T1D [7,20]. Furthermore, in this latter group, a decrease in the odds of being -DRB1*09 carriers was also found; however, this finding should be viewed with caution due to the large confidence interval that was observed. In the present study, PCA results suggested that patients who reported all relatives as being White showed a clear cluster.

Although the reported HLA system alleles’ effects on risk and protection for T1D varied across populations, the majority of studies carried out with patients with T1D were performed in homogeneous populations, mainly in European countries with predominant Caucasian ethnicity [4,20]. A study performed with African-Americans showed that HLA-DRB1*03 or -DRB1*04, considered to be European-derived alleles, and -DRB1*09, a possible African-derived [6] or Asian-derived allele [24,25], conferred higher risk for T1D. The role of this allele on susceptibility to T1D is still controversial. Some studies [6,24] have found a higher risk in heterozygotes carrying the allelic groups HLA-DRB1*03 or -DRB1*04, as did ours. Although the sample of relatives reported as being Yellow or Indigenous was small, patients who reported any relative as being Yellow had increased odds of being HLA-DRB1*09 carriers, but with a large confidence interval. These patients also reported having White relatives, and the majority were DRB1*03 and DRB1*09 carriers, without predominance. This fact was observed even in patients who reported all relatives as being Black, which probably indicates some degree of admixture with Europeans, Caucasians, among the ancestors beyond the 3rd generation [26]. Our findings showed that patients who reported all relatives as being Black still had the presence of HLA-DRB1*03. It is noteworthy that although African-Brazilians have more European ancestry than African-Americans [26,27], the risk alleles for T1D were the same. So far, most studies that were carried out with different ethnicities showed that HLA-DRB1*03 and/or -DRB1*04 were the most important risk alleles for T1D and did not include any relatives’ details [28,29,30,31]. For instance, a study carried out in India, with a very heterogeneous population composed of Indo-European and Dravidian ethnicities, showed that the presence of the DRB1*03 allele, followed by DRB1*04 allele, discriminated T1D from type 2 diabetes in young people of Indo-European ethnicity [30].

Considering that the aforementioned populations (Brazilian, African-American and Indian) have heterogeneous ethnicities with different degrees and types of admixture, and that DRB1*03 or DRB1*04 are probably European-derived alleles, our hypothesis is that the disease comes from European, Caucasian ethnicity. This fact could be related to the new concepts about the role of different immune systems, such as the HLA, that presented a high level of polymorphism and genetic variation worldwide, which could be linked to significant signals of human geographic expansion, demographic history, and cultural diversification [32,33]. In this context, tracing the ancient genetic history of each studied population could add information about the genetic origin of T1D in admixed populations. In addition, the detection of genetic profiles associated with T1D can help in immunological monitoring, the identification of factors that may contribute to the genetic risk for T1D, and proposing a specific treatment approach for population groups with different ancestral lineages.

Our study is the first multicenter study with patients having T1D that included a large multi-ethnic sample from all geographical regions of Brazil, and that also analyzed demographic data of a large number of participants’ relatives regarding birthplace and reported race/skin color, which added strength to our results. Another strength is that we used a uniform, standardized recruitment protocol in all participating centers, and the genotyping of three loci of the *HLA-DRB1, -DQA1* and -*DQB1* in all patients.

Our study has also some limitations that must be mentioned. We used only clinical criteria to define T1D. We did not measure autoantibodies against pancreatic islet β cells or serum C peptide levels, which could have led us to misclassify some cases as having T1D. However, the use of clinical criteria to define T1D is common in epidemiologic studies like ours [6]. All data regarding relatives were reported by the patients or their parents, which could make the data subject to error. Although reported skin color is considered a poor predictor of genomic ancestry in admixed populations, it gives an idea of the genetic structure of the Brazilian population [34]. In addition, it is important to highlight that the majority of epidemiologic studies carried out in Brazil have used self-reported race/skin color, which is therefore an important variable for comparing populations from different studies. Finally, although we have analyzed reported demographic data of patients’ relatives up to the 3rd generation, this time frame may not be enough to establish a relationship between the HLA system and the colonization of our country and its immigration history.

## 5. Conclusions

Our study showed that the most frequent alleles in patients with T1D were those that have already been described as risk alleles in multicenter studies conducted in Brazil and in other countries. The great majority of relatives of both genders were reported as being White, followed by Brown. Although no association between HLA system alleles with relatives’ birthplace was found and the majority of the relatives were born in Brazil, an increase in relatives born outside of Brazil was found from the 1st to the 3rd generation, with relatives coming mainly from the Iberian Peninsula and Italy, which reflects the history of Brazilian colonization.

Considering that most of our patients reported having a White relative, and that the most frequent allele and haplotype were DRB1*03:01 and 03:01~05:01g~02:01, respectively (probably European-Caucasian derived), we can link the ancestry of patients with T1D in Brazil back to European, Caucasian ethnicity. However, future studies with other genomic ancestry markers, both matrilineal and patrilineal, are needed to fill the knowledge gap regarding the genetic origin of T1D in admixed populations, such as the Brazilian.

## Figures and Tables

**Table 1:** Descriptive analysis of the study sample of Brazilian patients with type 1 diabetes (*n* = 662).

Variables	Mean ± SD	*n*	%
Gender			
Male		317	47.89
Female		345	52.11
Age, years	29.32 ± 12.52		
Age of diabetes diagnosis, years	14.5 ± 9.2		
Self-reported race/skin color (IBGE classification)			
White (Branca)		355	53.63
Black (Preta)		43	6.50
Brown (Parda)		255	38.52
Yellow (Amarela)		8	1.21
Indigenous (Indígena)		1	0.15
Birthplace (Brazilian geographic macro region)			
Southeast		367	55.44
South		53	8.01
Northeast		214	32.33
Midwest		2	0.30
North		26	3.93

SD = standard deviation. IBGE = Brazilian Institute of Geography and Statistics, race/skin color classification (Portuguese language).

**Table 2:** HLA-DRB1 -QA1 and -DQB1 allele’s frequencies in the study sample of Brazilian patients with type 1 diabetes.

HLA Haplotypes	*n*	%
HLA-DRB1*		
DRB1*01	110	8.31
DRB1*03	396	29.91
DRB1*04	400	30.21
DRB1*07	109	8.23
DRB1*08	45	3.40
DRB1*09	37	2.79
DRB1*10	13	0.98
DRB1*11	52	3.93
DRB1*12	9	0.68
DRB1*13	73	5.51
DRB1*14	15	1.13
DRB1*15	35	2.64
DRB1*16	30	2.27
HLA-DQA1*		
DQA1*01	266	20.12
DQA1*02	95	7.18
DQA1*03	452	34.19
DQA1*04	49	3.70
DQA1*05	458	34.64
DQA1*06	2	0.15
HLA-DQB1*		
DQB1*02	523	39.50
DQB1*03	492	37.31
DQB1*04	46	3.48
DQB1*05	167	12.61
DQB1*06	94	7.10

**Table 3:** Multiple logistic regression of predictors of HLA-DRB1* alleles in the study sample.

Variables	_adjusted_ OR	95% CI	*p* Value
HLA-DRB1*01 (Dependent variable)			
Data from relatives up to 3rd degree (Predictors)			
All relatives reported as white	1.35	0.83–2.21	0.219
All relatives reported as black	0.65	0.08–5.28	0.687
Any relative reported as indigenous	0.63	0.21–1.84	0.405
Any relative reported as yellow	–	–	–
Any relative born in Europe	0.84	0.50–1.43	0.536
HLA-DRB1*03 (Dependent variable)			
Data from relatives up to 3rd degree (Predictors)			
All relatives reported as white	1.53	1.04–2.24	0.028 *
All relatives reported as black	1.29	0.34–4.89	0.703
Any relative reported as indigenous	1.07	0.55–2.08	0.831
Any relative reported as yellow	1.03	0.25–4.19	0.960
Any relative born in Europe	0.85	0.58–1.25	0.427
HLA-DRB1*04 (Dependent variable)			
Data from relatives up to 3rd degree (Predictors)			
All relatives reported as white	1.37	0.93–2.01	0.105
All relatives reported as black	0.43	0.10–1.78	0.250
Any relative reported as indigenous	1.12	0.57–2.20	0.721
Any relative reported as yellow	0.29	0.05–1.47	0.136
Any relative born in Europe	0.85	0.57–1.25	0.411
HLA-DRB1*07 (Dependent variable)			
Data from relatives up to 3rd degree (Predictors)			
All relatives reported as white	0.36	0.19–0.67	0.001 *
All relatives reported as black	0.59	0.07–4.86	0.631
Any relative reported as indigenous	0.97	0.41–2.29	0.947
Any relative reported as yellow	1.59	0.31–8.08	0.571
Any relative born in Europe	1.51	0.90–2.54	0.117
HLA-DRB1*08 (Dependent variable)			
Data from relatives up to 3rd degree (Predictors)			
All relatives reported as white	0.55	0.22–1.39	0.211
All relatives reported as black	1.31	0.16–10.86	0.797
Any relative reported as indigenous	–	–	–
Any relative reported as yellow	3.51	0.68–18.10	0.132
Any relative born in Europe	0.39	0.13–1.16	0.093
HLA-DRB1*09 (Dependent variable)			
Data from relatives up to 3rd degree (Predictors)			
All relatives reported as white	0.16	0.03–0.72	0.017 *
All relatives reported as black	1.94	0.23–16.24	0.537
Any relative reported as indigenous	0.80	0.18–3.50	0.768
Any relative reported as yellow	9.35	2.10–41.55	0.003 *
Any relative born in Europe	1.35	0.55–3.28	0.506
HLA-DRB1*11 (Dependent variable)			
Data from relatives up to 3rd degree (Predictors)			
All relatives reported as white	0.34	0.13–0.86	0.023 *
All relatives reported as black	1.44	0.17–11.89	0.733
Any relative reported as indigenous	1.55	0.56–4.22	0.391
Any relative reported as yellow	–	–	–
Any relative born in Europe	1.56	0.78–3.14	0.206
HLA-DRB1*13 (Dependent variable)			
Data from relatives up to 3rd degree (Predictors)			
All relatives reported as white	1.14	0.62–2.07	0.664
All relatives reported as black	1.03	0.12–8.48	0.973
Any relative reported as indigenous	1.28	0.47–3.46	0.615
Any relative reported as yellow	–	–	–
Any relative born in Europe	0.89	0.48–1.66	0.733
HLA-DRB1*15 (Dependent variable)			
Data from relatives up to 3rd degree (Predictors)			
All relatives reported as white	0.81	0.32–2.03	0.661
All relatives reported as black	2.44	0.29–20.54	0.409
Any relative reported as indigenous	1.61	0.46–5.69	0.452
Any relative reported as yellow	–	–	–
Any relative born in Europe	1.15	0.48–2.74	0.744
HLA-DRB1*16 (Dependent variable)			
Data from relatives up to 3rd degree (Predictors)			
All relatives reported as white	1.01	0.39–2.59	0.983
All relatives reported as black	3.01	0.35–25.53	0.311
Any relative reported as indigenous	1.29	0.28–5.82	0.734
Any relative reported as yellow	3.44	0.39–29.65	0.260
Any relative born in Europe	1.13	0.44–2.88	0.785

OR = Odds ratio. 95% CI = 95% confidence interval. * *p* < 0.05.

**Table 4:** Distribution of HLA-DRB1* genotypes in Brazilian patients with type 1 diabetes who reported all parents, grandparents and great-grandparents as White (*n* = 164).

HLA-DRB1*/-DRB1* Genotype	*n*	%
03:01/03:01	16	9.76%
03:01/04:02	15	9.15%
03:01/04:04	13	7.93%
03:01/04:05	8	4.88%
03:01/04:01	6	3.66%
01:01/03:01	5	3.05%
03:01/13:02	5	3.05%
04:01/04:05	5	3.05%
03:01/07:01	4	2.44%
01:01/04:01	3	1.83%
01:01/04:05	3	1.83%
01:02/03:01	3	1.83%
03:01/08:01	3	1.83%
03:01/13:01	3	1.83%
03:01/16:01	3	1.83%
04:05/04:05	3	1.83%
01:01/04:04	2	1.22%
01:02/04:01	2	1.22%
03:01/04:03	2	1.22%
03:01/09:01	2	1.22%
04:01/04:02	2	1.22%
04:01/13:02	2	1.22%
04:02/04:02	2	1.22%
04:02/04:05	2	1.22%
04:05/10:01	2	1.22%
Other	48	<1%

## Data Availability

The data presented in this study are available on request from the corresponding author. The data are not publicly available due to privacy.

## Supplementary Materials

The following supporting information can be downloaded at: https://www.mdpi.com/article/10.3390/genes13060972/s1. Figure S1: Study sample flowchart. HLA, histocompatibility leukocyte antigen; T1D, type 1 diabetes; Figure S2: Distribution of HLA-DQA1 allele according to reported race-skin color of paternal relatives (a, b, c) and maternal relatives (d, e, f). Chi-square test was performed for statistical analysis; Figure S3: Distribution of HLA-DQB1 allele according to reported race-skin color of paternal relatives (a, b, c) and maternal relatives (d, e, f). Chi-square test was performed for statistical analysis; Figure S4: Distribution of HLA-DRB1 allele according to birthplace of 1st degree (a), 2nd degree (b), and 3rd degree relatives (c). Chi-square test was performed for statistical analysis; Figure S5: Distribution of carriers of HLA-DRB1*03 or/and *04 alleles according to birthplace of the 1st degree (a), 2nd degree (b), and 3rd degree relatives (c). Chi-square test was performed for statistical analysis; Figure S6: Principal component analysis of the presence of HLA-DRB1* alleles in Brazilian patients with type 1 diabetes by race-skin color of relatives: only White (a), only Black (b), any Indigenous (c), and any Yellow (d); Table S1: Distribution of reported race-skin color data according to paternal/maternal lineage; Table S2: Descriptive analysis from reported birthplace data; Table S3: Distribution of HLA-DRB1 alleles in the study sample of Brazilian patients with type 1 diabetes; Table S4: Distribution of HLA-DQA1 allele in Brazilian patients with type 1 diabetes; Table S5: Distribution of HLA-DQB1 alleles in Brazilian patients with type 1 diabetes; Table S6: Distribution of HLA-DRB1*/DRB1*genotype in Brazilian patients with type 1 diabetes; Table S7: Distribution of HLA-DRB1* genotypes in Brazilian patients with type 1 diabetes who reported relatives as black (up to 3rd degree relatives); Table S8: Distribution of HLA-DRB1* genotypes in Brazilian patients with type 1 diabetes who reported any relatives (up to 3rd degree) as yellow (*n* = 8) or indigenous (*n* = 38); Table S9: Distribution of HLA–DRB1*~DQA1*~DQB1* haplotypes in the study sample.
