# Supplementary material for: HLA Genotypes and Type 1 Diabetes and Its Relationship to Reported Race/Skin Color in Their Relatives: A Brazilian Multicenter Study
Source: Genes (Basel). 2022 May 29;13(6):972. doi: 10.3390/genes13060972 (PMC9223352; doi:10.3390/genes13060972)
Supplement: Supplementary file 1 [file genes-13-00972-s001.zip › genes-1733185-supplementary.pdf]

Supplementary Material

HLA genotypes and type 1 diabetes and its relationship to reported race/skin color in their relatives: a Brazilian's multicenter study

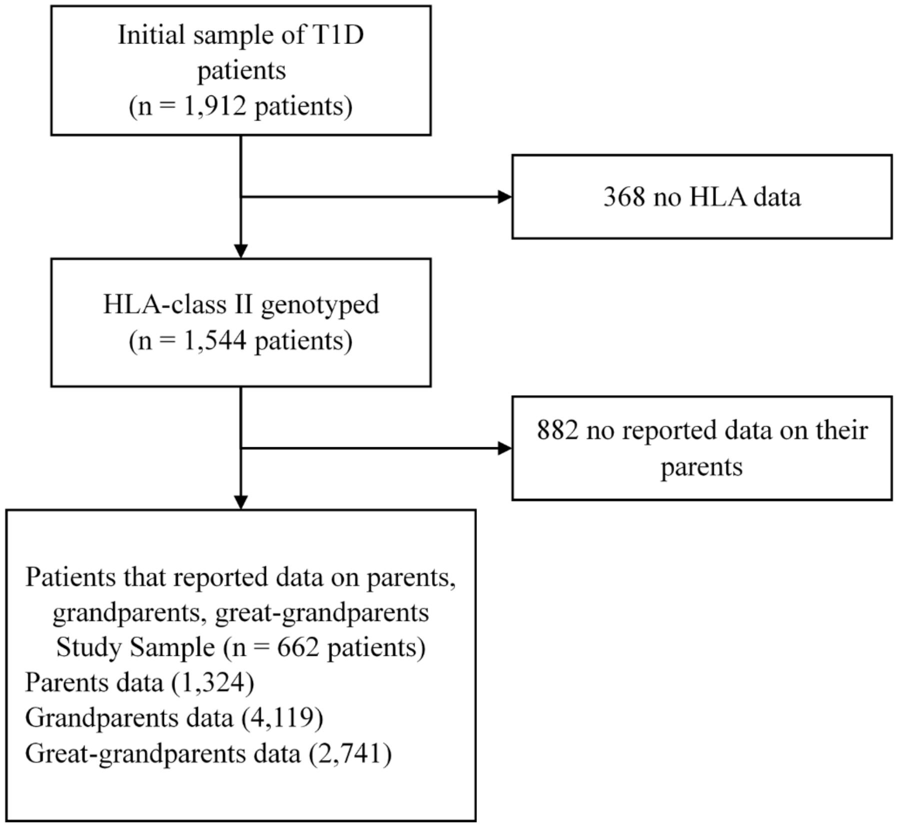

Supplementary Figure S1. Study sample flowchart. HLA, histocompatibility leukocyte antigen; T1D, type 1 diabetes.

Supplementary Table S1. Distribution of reported race-skin color data according to paternal/maternal lineage.

Paternal lineage reported race-skin color (n = 662)	Father	Paternal grandfather	Paternal grandmother
	n (%)	n (%)	n (%)
White	364(54.98)	320(48.34)	355(53.63)
Black	62(9.37)	61(9.21)	46(6.95)
Brown	229(34.59)	162(24.47)	170(25.68)
Yellow	4(0.60)	2(0.30)	2(0.30)
Indigenous	3(0.45)	5(0.76)	7(1.06)
No data	-	112(16.92)	82(12.39)
**Maternal lineage reported race-skin color (n = 662)**	**Mother**	**Maternal grandfather**	**Maternal grandmother**
	n (%)	n (%)	n (%)
White	374(56.50)	339(51.21)	397(59.97)
Black	48(7.25)	78(11.78)	55(8.31)
Brown	237(35.80)	185(27.95)	171(25.83)
Yellow	3(0.45)	1(0.15)	1(0.15)
Indigenous	0(0)	7(1.06)	9(1.36)
No data	-	52(7.85)	29(4.38)

Supplementary Table S2. Descriptive analysis from reported birthplace data.

Variables	Relatives, up to 3rd degree (n=662)				
	1st degree	2nd degree	3rd degree	Male	Female
	n (%)	n (%)	n (%)	n (%)	n (%)
Born location					
Born in Brazil	654 (98.79)	584 (88.22)	503 (75.98)	510 (77.04)	534 (80.66)
Born outside of Brazil	8 (1.21)	78 (11.78)	159 (24.02)	152 (22.96)	128 (19.34)
Location outside of Brazil					
Iberian Peninsula	5 (0.76)	44 (6.65)	83 (12.53)	79 (11.93)	64 (9.66)
Italy	2 (0.30)	23 (3.47)	61 (9.21)	55 (8.30)	42 (6.34)
Caucasus region	1 (0.15)	9 (1.36)	22 (3.32)	17 (2.56)	17 (2.56)
Japan	-	4 (0.60)	4 (0.60)	4 (0.60)	4 (0.60)
Middle East	-	1 (0.15)	2 (0.30)	2 (0.30)	2 (0.30)
Africa	-	-	1 (0.15)	1 (0.15)	1 (0.15)

Supplementary Table S3. Distribution of HLA-DRB1 alleles in the study sample of Brazilian patients with type 1 diabetes.

HLA-DRB1* alleles	n	%
01:01	58	4.38%
01:02	48	3.63%
01:03	4	0.30%
03:01	381	28.78%
03:02	12	0.91%
03:05	1	0.08%
03:06	1	0.08%
03:12	1	0.08%
04:01	88	6.65%
04:02	85	6.42%
04:03	4	0.30%
04:04	64	4.83%
04:05	128	9.67%
04:07	11	0.83%
04:08	2	0.15%
04:09	3	0.23%
04:10	2	0.15%
04:11	13	0.98%
07:01	108	8.16%
07:02	1	0.08%
08:01	22	1.66%
08:02	8	0.60%
08:03	1	0.08%
08:04	9	0.68%
08:07	5	0.38%
09:01	37	2.79%
10:01	13	0.98%
11:01	35	2.64%
11:02	6	0.45%
11:03	2	0.15%
11:04	9	0.68%
12:01	9	0.68%
13:01	27	2.04%
13:02	37	2.79%
13:03	8	0.60%
13:04	1	0.08%
14:01	4	0.30%
14:02	7	0.53%
14:04	1	0.08%
14:06	1	0.08%
14:54	2	0.15%
15:01	17	1.28%
15:03	17	1.28%
15:44	1	0.08%
16:01	17	1.28%
16:02	13	0.98%

Supplementary Table S4. Distribution of HLA-DQA1 allele in Brazilian patients with type 1 diabetes.

HLADQA1* alleles	n	%
01:01	140	10.59%
01:02	104	7.87%
01:03	22	1.66%
02:01	94	7.11%
02:02	1	0.08%
03:01	452	34.19%
04:01	48	3.63%
04:03	1	0.08%
05:01	453	34.27%
05:03	5	0.38%
06:01	2	0.15%

Supplementary Table S5. Distribution of HLA-DQB1 alleles in Brazilian patients with type 1 diabetes.

HLA-DQB1* alleles	n	%
02:01	360	27.19%
02:02	158	11.93%
02:03	5	0.38%
03:01	79	5.97%
03:02	386	29.15%
03:03	23	1.74%
03:19	4	0.30%
03:40/03:110/03:141/03:155	2	0.15%
04:02	46	3.47%
05:01	130	9.82%
05:02	24	1.81%
05:03	7	0.53%
05:07	6	0.45%
06:01	2	0.15%
06:02	34	2.57%
06:03	23	1.74%
06:04	25	1.89%
06:09	9	0.68%
06:10	1	0.08%
16:02	13	0.98%

Supplementary Table S6. Distribution of HLA-DRB1*/DRB1*genotype in Brazilian patients with type 1 diabetes.

HLA-DRB1*/DRB1* genotype	n	%
DRB1*03/DRB1*04	156	23.56
DRB1*03/DRB1*03	58	8.76
DRB1*04/DRB1*04	44	6.65
DRB1*01/DRB1*04	39	5.89
DRB1*01/DRB1*03	30	4.53
DRB1*03/DRB1*07	28	4.23
DRB1*04/DRB1*07	28	4.23
DRB1*04/DRB1*13	26	3.93
DRB1*04/DRB1*11	17	2.57
DRB1*03/DRB1*13	16	2.42
DRB1*04/DRB1*08	13	1.96
DRB1*03/DRB1*16	12	1.81
DRB1*07/DRB1*08	11	1.66
DRB1*07/DRB1*13	11	1.66
DRB1*03/DRB1*08	9	1.36
DRB1*03/DRB1*09	9	1.36
DRB1*04/DRB1*16	9	1.36
DRB1*03/DRB1*11	8	1.21
DRB1*01/DRB1*09	7	1.06
DRB1*01/DRB1*13	7	1.06
DRB1*04/DRB1*09	7	1.06
DRB1*04/DRB1*10	7	1.06
Other	110	< 1%

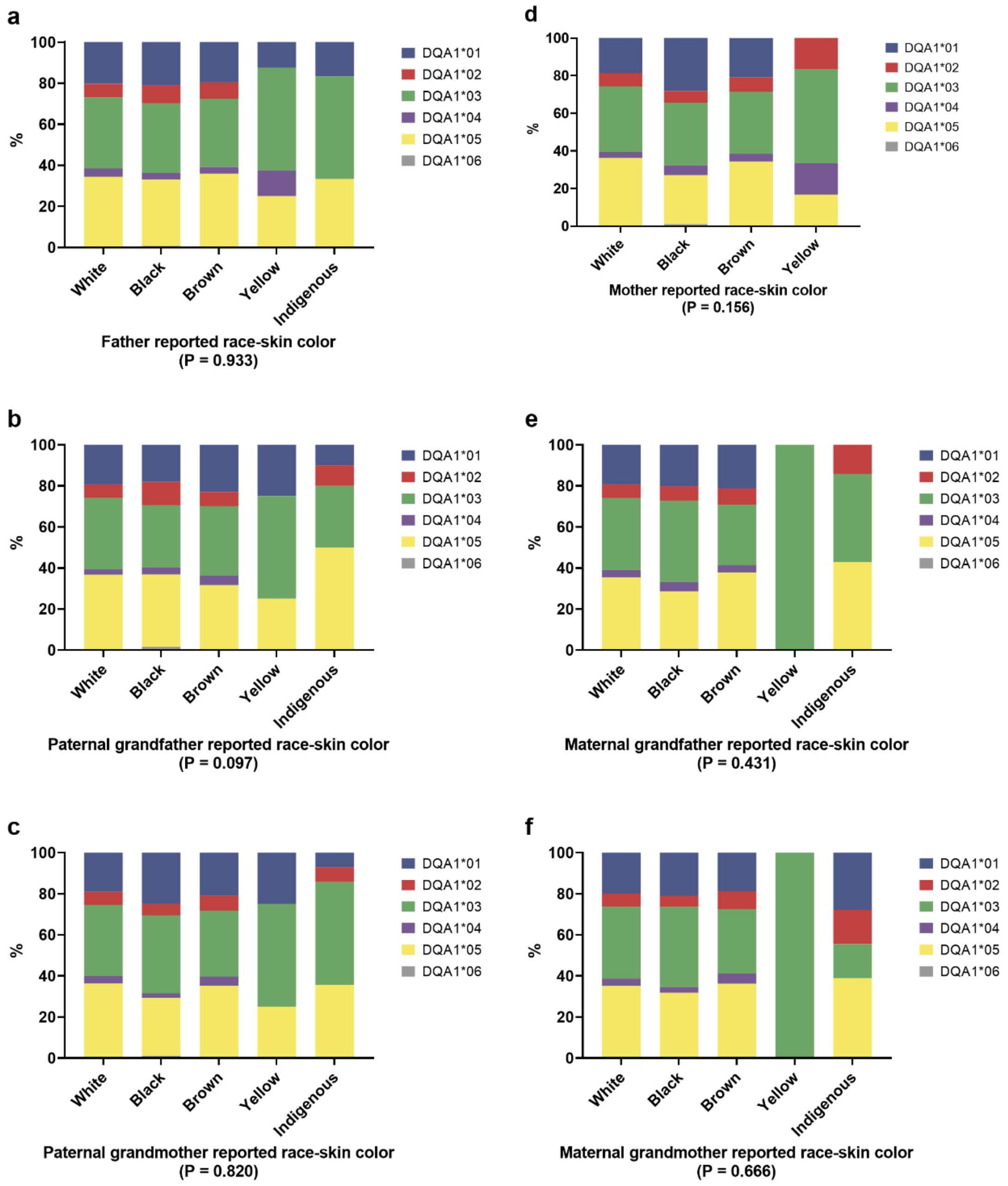

Supplementary Figure S2. Distribution of HLA-DQA1 allele according to reported race-skin color of paternal relatives (a, b, c) and maternal relatives (d, e, f). Chi-square test was performed for statistical analysis.

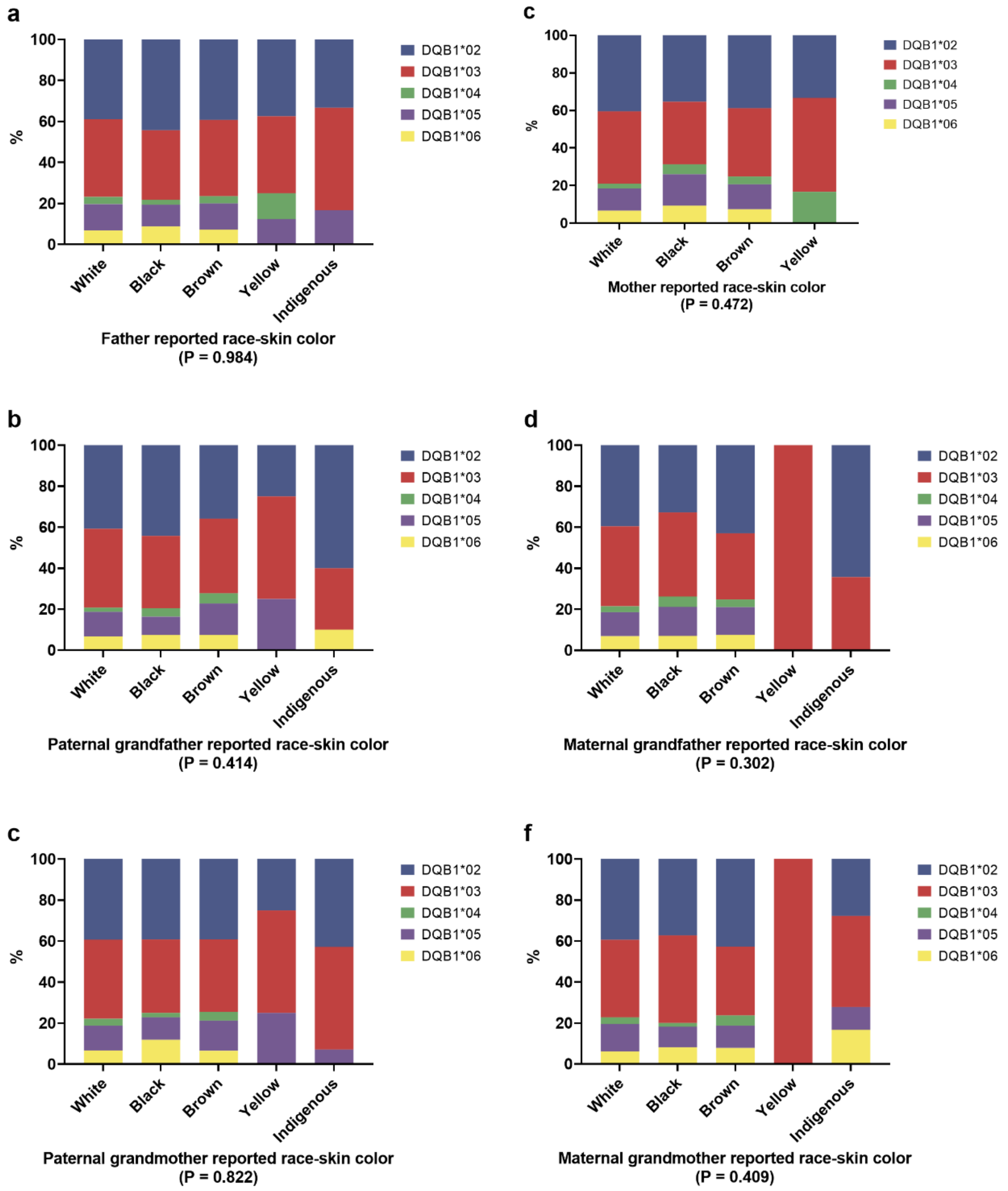

Supplementary Figure S3. Distribution of HLA-DQB1 allele according to reported race-skin color of paternal relatives (a, b, c) and maternal relatives (d, e, f). Chi-square test was performed for statistical analysis.

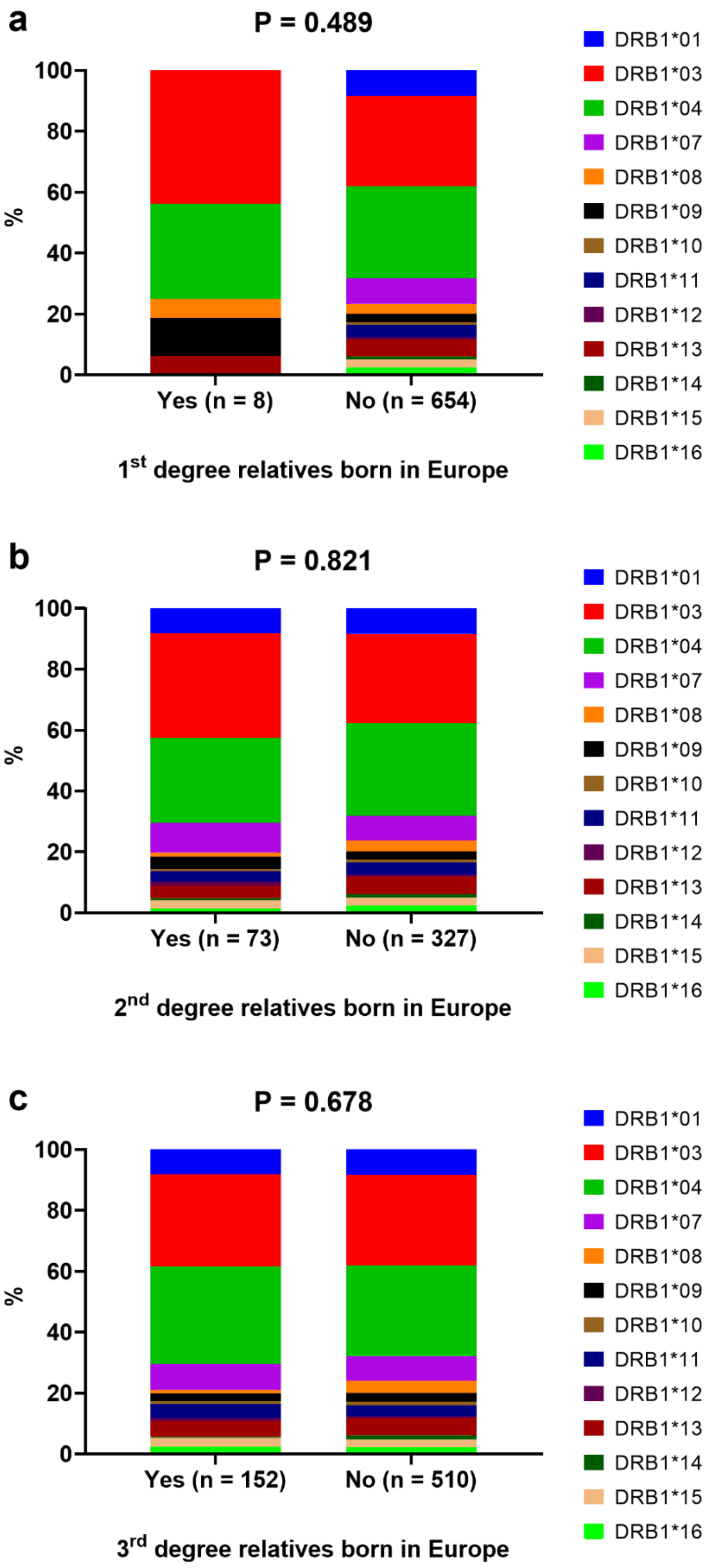

Supplementary Figure S4. Distribution of HLA-DRB1 allele according to birthplace of 1st degree (a), 2nd degree (b), and 3rd degree relatives (c). Chi-square test was performed for statistical analysis.

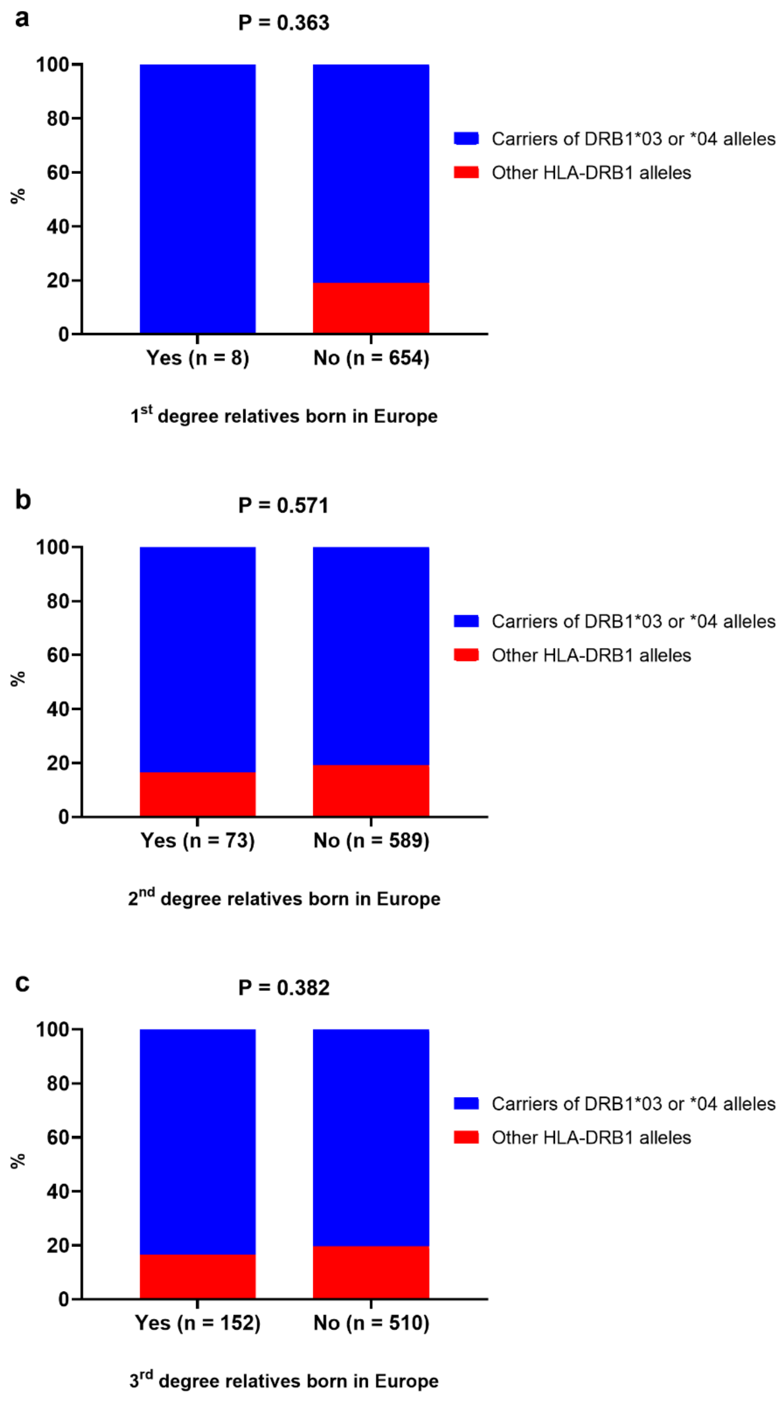

Supplementary Figure S5. Distribution of carriers of HLA-DRB1*03 or/and *04 alleles according to birthplace of the 1st degree (a), 2nd degree (b), and 3rd degree relatives (c). Chi-square test was performed for statistical analysis.

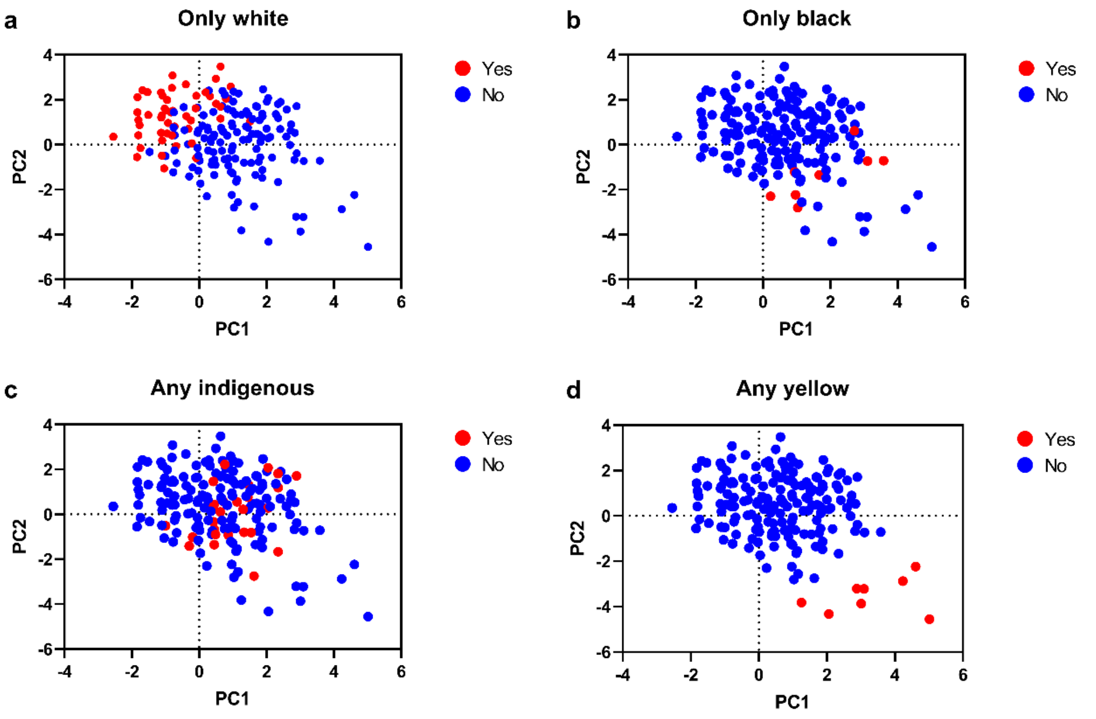

Supplementary Figure S6. Principal component analysis of the presence of HLA-DRB1* alleles in Brazilian patients with type 1 diabetes by race-skin color of relatives: only White (a), only Black (b), any Indigenous (c), and any Yellow (d).

Supplementary Table S7. Distribution of HLA-DRB1* genotypes in Brazilian patients with type 1 diabetes who reported relatives as black (up to 3rd degree relatives).

HLA-DRB1*/-DRB1* genotype	n	%
Patients who reported any black (up to 3rd degree relatives) (n = 186)		
03:01/03:01	14	7.53%
03:01/04:04	11	5.91%
03:01/04:05	9	4.84%
03:01/04:02	7	3.76%
03:01/04:01	6	3.23%
04:02/04:05	5	2.69%
01:01/04:05	4	2.15%
03:01/07:01	4	2.15%
07:01/08:01	4	2.15%
01:02/04:05	3	1.61%
03:01/11:01	3	1.61%
03:01/16:02	3	1.61%
03:02/04:02	3	1.61%
04:01/07:01	3	1.61%
07:01/07:01	3	1.61%
01:01/03:01	2	1.08%
01:02/03:01	2	1.08%
01:02/09:01	2	1.08%
03:01/04:07	2	1.08%
03:01/08:01	2	1.08%
03:01/09:01	2	1.08%
03:01/14:02	2	1.08%
03:01/16:01	2	1.08%
04:01/08:07	2	1.08%
04:01/11:01	2	1.08%
04:02/07:01	2	1.08%
04:02/09:01	2	1.08%
04:04/04:05	2	1.08%
04:04/07:01	2	1.08%
04:05/04:05	2	1.08%
04:05/07:01	2	1.08%
07:01/08:04	2	1.08%
07:01/09:01	2	1.08%
07:01/11:01	2	1.08%
07:01/13:02	2	1.08%
09:01/13:02	2	1.08%
11:01/11:01	2	1.08%
Other	60	<1%
Patients who reported only black (up to 3rd degree relatives) (n = 9)		
01:01/15:03	1	11.11%
03:01/03:01	1	11.11%
03:01/04:02	1	11.11%
03:01/04:07	1	11.11%
03:01/11:01	1	11.11%
03:01/16:02	1	11.11%
04:01/04:05	1	11.11%
07:01/08:01	1	11.11%
09:01/13:02	1	11.11%

Supplementary Table S8. Distribution of HLA-DRB1* genotypes in Brazilian patients with type 1 diabetes who reported any relatives (up to 3rd degree) as yellow (n = 8) or indigenous (n = 38).

HLA-DRB1*/-DRB1* genotype	n	%
Patients who reported any indigenous (up to 3rd degree relatives) (n = 38)		
01:02/03:01	1	2.63%
01:02/04:02	1	2.63%
01:02/13:02	1	2.63%
01:02/13:03	1	2.63%
03:01/03:01	2	5.26%
03:01/04:01	2	5.26%
03:01/04:02	3	7.89%
03:01/04:04	2	5.26%
03:01/04:05	1	2.63%
03:01/04:11	2	5.26%
03:01/07:01	2	5.26%
03:01/09:01	1	2.63%
03:01/11:02	1	2.63%
03:01/16:01	1	2.63%
03:02/14:06	1	2.63%
04:01/15:03	1	2.63%
04:02/04:05	1	2.63%
04:02/13:02	1	2.63%
04:02/16:01	1	2.63%
04:04/09:01	1	2.63%
04:05/07:01	1	2.63%
04:05/11:01	2	5.26%
04:05/13:03	1	2.63%
04:09/07:01	1	2.63%
07:01/07:01	1	2.63%
07:01/11:01	1	2.63%
07:01/13:02	1	2.63%
10:01/15:03	1	2.63%
11:04/15:01	1	2.63%
14:02/14:02	1	2.63%
Patients who reported any yellow (up to 3rd degree relatives) (n=8)		
03:01/04:01	1	12.50%
03:01/08:02	1	12.50%
03:01/16:02	1	12.50%
03:02/07:01	1	12.50%
04:04/09:01	1	12.50%
07:01/08:02	1	12.50%
09:01/12:01g	1	12.50%
09:01/14:04	1	12.50%

Supplementary Table S9. Distribution of HLA–DRB1*~DQA1*~DQB1* haplotypes in the study sample.

Haplotype	n	%
03:01~05:01g~02:01	219	16.54%
04:05~03:01g~03:02	72	5.44%
03:01~03:01g~02:01	71	5.36%
07:01~02:01~02:02	52	3.93%
04:02~03:01g~03:02	50	3.78%
04:01~03:01g~03:02	48	3.63%
Other (<3%)	812	61.32%
